# Main Quality Attributes of Monoclonal Antibodies and Effect of Cell Culture Components

**DOI:** 10.18869/acadpub.ibj.21.3.131

**Published:** 2017-05

**Authors:** Fatemeh Torkashvand, Behrouz Vaziri

**Affiliations:** Biotechnology Research Center, Pasteur Institute of Iran, Tehran, Iran

**Keywords:** Main quality attributes, Monoclonal antibodies, Glycosylation, Fragmentation

## Abstract

The culture media optimization is an inevitable part of upstream process development in therapeutic monoclonal antibodies (mAbs) production. The quality by design (QbD) approach defines the assured quality of the final product through the development stage. An important step in QbD is determination of the main quality attributes. During the media optimization, some of the main quality attributes such as glycosylation pattern, charge variants, aggregates, and low-molecular-weight species could be significantly altered. Here, we provide an overview of how cell culture medium components affect the main quality attributes of the mAbs. Knowing the relationship between the culture media components and the main quality attributes could be successfully utilized for rational optimization of mammalian cell culture media for industrial mAbs production.

## INTRODUCTION

Process development is a state-of-the-art method to establish a manufacturing line in biopharmaceuticals. Although a significant shift in productivity is the main goal of process development, achieving appropriate quality attributes is also of great concern[1]. Quality by design (QbD) is a new approach to develop and to manufacture pharmaceutical products. QbD guarantees product quality and ensures that a consistent product with preferred quality attributes is generated[2,3]. Regulatory agencies encourage its application in the manufacture of all new pharmaceuticals containing biological products[2-4].

The cell line and its recombinant DNA construct, culture media, and process conditions are three important parameters that influence recombinant protein quality properties in the manufacture of biopharmaceuticals. The culture media and the control of process conditions are very important in process development[5,6]. In fact, the cell metabolism directly depends on the culture conditions, including the pH[7], the temperature[8], the oxygen tension[7], the CO_2_ amount in the culture broth[9], and also the mode of processing, i.e., perfusion or fed-batch mode[10]. Diverse metabolic outcomes states that result from modifications in these culture parameters might produce proteins with altered quality attributes. Many review articles have been published in this field with a focus on the cell line[11-13] and cell culture parameters[14,15]. Moreover, with concentration on the regulation of certain media constituents and by supplementing the medium with specific co-factors, the glycosylation profile[15], the charge variants[16], the aggregation amount[17,18] and the level of low-molecular-weight (LMW) variants[19] can be controlled.

At this time, monoclonal antibodies (mAbs) are the main products in the pipeline of the biopharmaceutical industry. Numerous studies have reported different impacts of glycosylation, charge variants, aggregates, and fragments on the biological activity and pharmacokinetics[20-23]. The purpose of this review is to discuss the main quality attributes of mAbs that can be changed directly by culture conditions, and to review the culture conditions and culture media components that affect these attributes.

### Glycosylation

#### Importance

Glycosylation is a complicated process of the attachment of oligosaccharides to the polypeptide backbone of a protein, which occurs in the endoplasmic reticulum and Golgi apparatus. There are two main kinds of glycosylation[24]: asparagine (Asn)-linked glycosylation or N-linked glycosylation, and serine/threonine-O-linked glycosylation. In mAbs, the Asn-linked glycosylation is the most common[25]. The N-glycans are linked to the two conserved Asn residues (Asn 297) in the CH2 domain of the Fc region[26]. The presence or absence of certain oligosaccharides can affect mAb stability[27], *in vivo* efficacy[27-29], antibody-dependent cell-mediated cytotoxicity (ADCC)[27,30], complement-dependent cytotoxicity (CDC) activities[31], pharmacokinetics[22], clearance rate[1], and immunogenicity[1,32]. Hence, the precise control of glycosylation of mAbs is critical.

#### 2-2-N-glycosylation types

**Fig. 1:**
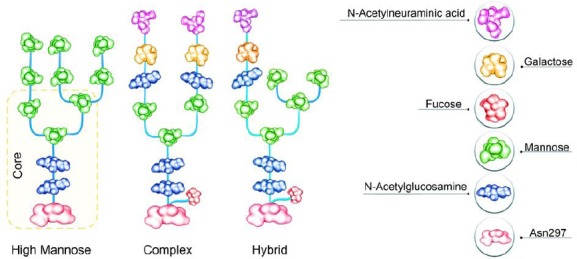
The schematic representation of the composition of different groups of N-glycans containing high mannose, complex, and hybrid types.

**Fig. 2:**
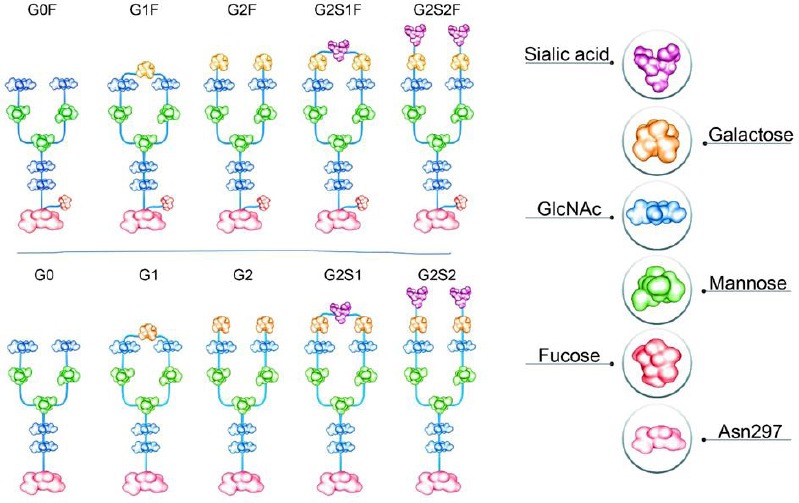
The schematic representation of major N-linked glycoforms of mAb therapeutics. G0: asialo, agalactose, biantennary complex (common core [Man3GlcNAc2] with terminal two GlcNAc residues), G0F: asialo, agalactose, biantennary complex, core substituted with fucose, G1: asialo, mono-galactosylated, biantennary complex, G1F: asialo, mono-galactosylated, biantennary complex, core substituted with fucose, G2F: asialo, galactosylated, biantennary complex, core substituted with fucose. G, galactose; S, sialo (sialic acid)

In the endoplasmic reticulum, the oligosaccharide chain is attached to the protein backbone and consequently forms an oligomannose species through a series of enzymatic reactions. In mammalian cells, the glycoprotein undergoes further processing in the Golgi[14,27]. N-glycans can be classified into three groups, which have a shared core comprising two N-acetylglucoseamine (GlcNAc) residues and three mannose types in a branched form (Fig. 1). The different groups are:

1) The high-mannose (HM) type that comprises only mannose residues attached to the core. While the HM amount on the endogenous human IgG is usually very low, the HM amount of the recombinant mAbs can range from 1% to ≥20%. Due to the quicker serum clearance rate of HM glycans compared to other Fc-glycans, the pharmacokinetic properties of these mAbs are affected[33,34]. Additionally, the HM glycoforms are concomitant with enhanced ADCC activity[34,35]. Therefore, the HM amount of mAbs can be considered to be an important quality attribute in the production process.
2) The complex type containing different kinds of monosaccharide in their antennal region (Fig. 2). Galactose amount may influence CDC, and the sialylation amount may influence functionality or inflammatory characteristics[15]. The lack of core-fucosylation results in enhanced ADCC[7]. For instance, non-fucosylated mAbs display fiftyfold to thousandfold higher efficacy than their fucosylated counterparts[30].
3) The hybrid type, which has properties from both HM and complex types attached to the core.

#### Glycosylation during cell culture

It is understood that differences in the N-linked glycan profile can take place during the mAb production process[7,36]. The cell culture conditions containing culture media elements, the accessibility of the nucleotide sugar substrates, the expression amounts of the enzymes involved in the attachment, and the transformation of carbohydrate structures determine the amount of antennarity and sialylation[14].

Manganese plays an important role in the glycosylation pathway[15,37,38]. As a co-factor of many enzymes, manganese controls the glycosylation profile[38]. It has been shown that increased nucleotide-sugar precursors levels, comprising UDP (uridine diphosphate)-Hex, UDP-HexNAc, and cytidine monophosphate-sialic acid, enhance the glycosylation of mAbs[39].

It has been shown that the glucose limitation in culture medium can lead to a reduced UDP GlcNAc availability[40] which in turn results in glycosylation heterogeneity[41]. In a Chinese hamster ovary (CHO) cell culture experiment, it was seen that the amount of non-glycosylated antibody was correlated to the extent of time the cells deprived of glucose[42]. In a different study in fed-batch culture mode, with the human cell line rF2N78, it has been shown that due to the lack of glucose in the feed, nearly 44% of the product was aglycosylated. No aglycosylated antibody was expressed when glucose was fed throughout the culture[43]. There are reports that glucose and glutamine (Gln) concentrations below 1 mM were harmful to glycosylation[29,44,45]. Also, variations in other cell culture conditions such as dissolved oxygen, bioreactor pH, ammonia, and shear stress, have been shown to affect the glycosylation of therapeutic mAbs. Their terminal galactosylation may be affected by such variations[14]. The variable presence of terminal galactose residues leads to the heterogeneity of Rituximab glycosylation[15,46]. The effect of Rituximab terminal galactose residues on CDC activity originates from the involvement of galactose residues in the binding of Rituximab to complement C1q[46]. Therefore, the agalactose form of Rituximab is considered as a serious impurity.

### Analytical methods for the detection of mAb glycosylation

**Fig. 3:**
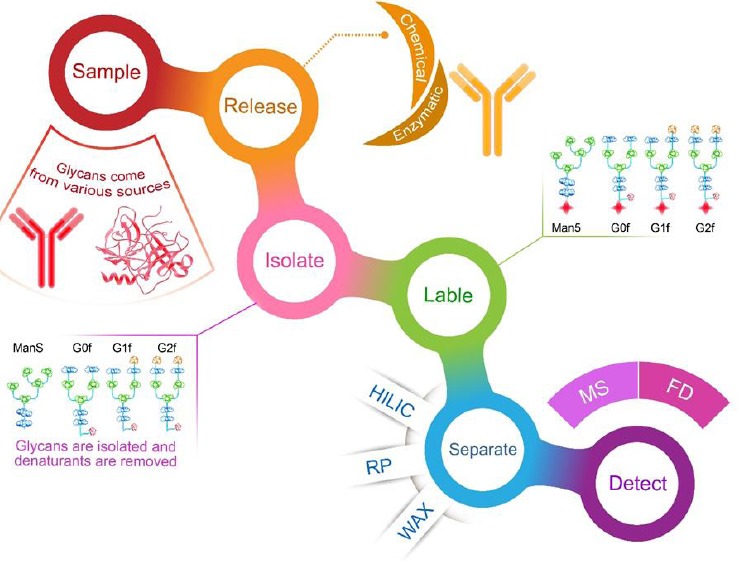
Workflow of glycan profiling, hydrophilic interaction chromatography (HILIC), reverse-phase chromatography (RP), weak anion exchange chromatography (WAX), mass spectrometry (MS), and fluorescence detector (FD). G0: asialo, agalactose, biantennary complex (common core [Man3GlcNAc2] with terminal two GlcNAc residues), G0F: asialo, agalactose, biantennary complex, core substituted with fucose, G1: asialo, mono-galactosylated, biantennary complex, G1F: asialo, mono-galactosylated, biantennary complex, core substituted with fucose, G2F: asialo, galactosylated, biantennary complex, core substituted with fucose. G, galactose

Several analytical methods are used to analyze glycosylation. Some of those are nuclear magnetic resonance, mass spectrometry, high performance liquid chromatography (HPLC), and capillary electrophoresis (CE). The most frequently used quantitative tools to analyze glycosylation are HPLC and CE. HPLC is used either with fluorescence detection[47-49] or with pulsed amperometric detection[50,51] and CE with a laser-induced fluorescence detector for various fluorescently-labelled glycans[52]. In HPLC-based methods, in the first step, glycans are released by chemical or enzymatic methods. The second step is the separation of the released glycans and the sample clean-up for the elimination of salts or denaturants. Labelling with appropriate reagents is done to improve detection. Then chromatographic techniques are used to separate the released, purified, and labelled or unlabelled glycans[53]. The common separation-based techniques that are used for the characterization of mAb glycoproteins are reverse-phase HPLC, hydrophilic interaction chromatography, and high-performance anion-exchange chromatography (Fig. 3).

### Charge variants

Recombinant mAbs undergo chemical degradation through diverse mechanisms comprising deamidation, oxidation, isomerization, and fragmentation that result in several charge variants and heterogeneity formation, consequently modifying their pI values[20].

#### Importance

**Fig. 4:**
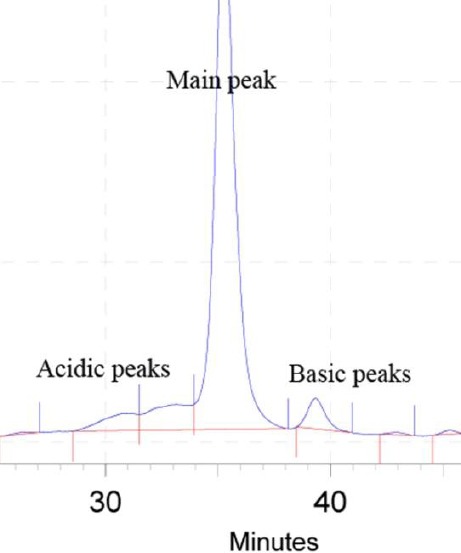
The cation exchange chromatogram representing different charge variants containing acidic, main, and basic peaks. The chromatogram is related to a homemade monoclonal antibody.

The analysis of charge heterogeneity in the mAbs characterization is essential because it provides significant information about product quality and stability[54]. Charge variants with a relatively lower pI are mentioned as acidic variants, while charge variants with a relatively higher pI are mentioned as basic variants (Fig. 4). Charge variants may significantly influence the *in vitro* and *in vivo* properties of antibodies. It has been revealed that they can change the binding to proteins or cell membrane targets, thereby affecting the tissue penetration, tissue distribution, and pharmacokinetics of the antibodies[20,55-58]. There is enough evidence in the literature to recommend that the existence of acidic species variants on mAbs can at least have an effect on the resulting protein’s efficacy and function[59-61]. The impacts of the charge variants depend highly on the nature, site, and the amount of post-translational modifications that cause the acidic and basic variants’ formation[62]. Therefore, mAb charge variant levels must be controlled exactly. At present, little information is available on the control of these variants using process parameters.

#### Charge variants types

Main species

The main peak of charge variant chromatograms usually contains species with three kinds of usual post-translational modifications: (1) Cyclization of the N-terminal Gln to pyroGlu, (2) elimination of the heavy chain C-terminal lysine (Lys), and (3) glycosylation of the conserved Asn residue in the CH2 domain with neutral oligosaccharides. At the time of analysis, most of the antibodies comprised N-terminal pyroGlu instead of the original Gln, and therefore elute as the main peak[63,64]. Antibodies without any C-terminal Lys are typically observed in the main species[63-67]. The preserved Asn residue in the CH2 domain is glycosylated. The core-fucosylated complex bi-antennary structures with zero, one, or two terminal galactose residues are the main glycoforms of recombinant mAbs from mammalian cell cultures[68].

Acidic species

Charge variants with a relatively lower pI are termed acidic variants. Table 1 summarizes the central reasons for the formation of acidic species. The major cause of acidic species formation, which has been extensively reported, is deamidation of Asn residues. Deamidation happens both in the variable domains, particularly complementarity-determining regions, as well as in the constant domains. Deamidation of Asn residues in the complementarity-determining regions always leads to the acidic species formation[63,69-73].

**Table 1:** The modifications that form acidic variants

Number	Acidic variants	Basic variants
1	Deamidation	N-terminal Glu
2	Non-classical disulfide linkage	Isomerization of Asp
3	Trisulfide bonds	Met oxidation
4	Glycation	C-terminal Lys
5	High mannose	Incomplete disulfide bonds
6	Sialic acid	Amidation
7	Thiosulfide modification	Succinimide
8	Cysteinylation	Mutation from Ser to Arg
9	Non-reduced species	Aggregates
10	Reduced disulfide bonds	Fragments
11	Modification by maleuric acid	Aglycosylation
12	Fragments	Incomplete removal of leader sequence

Basic species

Charge variants with a relatively higher pI are basic variants. Table 1 summarizes the main reasons for the basic species generation. The major cause for the generation of basic species is incomplete removal of C-terminal Lys. Due to the further positive charges, mAbs with heavy chain C-terminal Lys are more basic than the main species[20,63-65,68,74-78].

#### Charge variants during cell culture

The culture temperature displays important effects on mAb charge variants’ distribution[79]. Reducing the temperature and accelerating the temperature shift time considerably decrease the acidic charge variant amount[61]. In a study, Zhang *et al*.[79] showed that decreasing the culture temperature enhanced the Lys variant amount, which can be the main reason for the increased basic variant amount, also they showed that cultivations at sub-physiological temperatures in both batch and fed-batch culture modes reduced the mAb acidic variant levels, but the basic ones were enhanced. It can be related to the reduction of carboxypeptidase B transcription level. However, the mechanism by which a temperature downshift decreases the acidic charge variants’ level has not been clarified yet.

There was a straight correlation between the proline amidation level and the basic peak level. Kaschak *et al*.[54] observed that the proline amidation was sensitive to copper ion concentration in the culture medium during cell culture. They showed that a higher Cu^2+^ ion concentration results in the higher level of proline amidation. They also showed that if the copper concentration increases and the zinc concentration decreases in a chemically defined medium, the level of C-terminal Lys variants will enhance[16]. Deamidation modification in target mAb is decreased by glycerol and sodium chloride[80] and increased by iron concentration enhancment[81]. Increase in the sodium butyrate concentration in CHO cell culture medium enhances mAbs basic charge variants[82]. Moreover, it has been found that the supplementation of mammalian cell culture media with the bioflavonoid chemical family can decrease acidic species of recombinant mAbs[83].

#### Analytical methods for the detection of mAb charge variants

Several methods are used to detect charge variants of recombinant mAbs. These include isoelectric focusing gel electrophoresis[71,74,84], capillary isoelectric focusing electrophoresis[76,85], and cation[71,74,76,85-87] and anion[84,87] exchange chromatography.

### Fragmentation and aggregation

#### Importance

Protein aggregation and fragmentation may lead to immunogenicity, loss of biological activity, and other side-effects[88-92]. These modifications are host cell line, clone, and process-dependent[15,93].

#### Fragmentation

Fragmentation is a common type of degradation and can be attributed to the disruption of a covalent peptide bond. It may take place spontaneously or by enzymatic reaction[92]. To evaluate the purity and integrity of the target protein, it is necessary to monitor the mAbs fragmentation as a critical quality attribute. The fragmentation pattern of mAb denotes a fingerprint of stability and production consistency.

#### Aggregation

In the manufacture of therapeutic proteins, aggregation is a common problem. Protein aggregates can be categorized in several ways, including soluble/insoluble, covalent/non-covalent, reversible/non-reversible, and native/denatured[18,94,95]. These structural changes are significant because they can cause a loss of activity of the intact proteins. Furthermore, aggregation and misfolding can induce a new epitope presentation, leading to an adverse immune response[88,96]. The control and avoidance of aggregation in the manufacturing process are needed because aggregates affect drug performance and safety[97,98].

#### Aggregate formation during mAb manufacturing processes

Physicochemical stresses, such as changes in the osmolality and pH of the medium, or changes in the culture temperature, protein concentration, oxygen and shear forces can aggregate the secreted proteins[94,99]. Stresses to the protein, such as freezing contact with air, or interactions with metal surfaces, may lead to undesired post-translational modification, which result in aggregates formation. Mechanical stresses may lead to protein aggregation[100-102]. Osmolytes in the form of small organic components, such as sugars, polyols, and amino acids help as chemical chaperones to stabilize proteins and stop aggregation[103-105].

#### Fragmentation during cell culture

Fragmentation may occur because of the action of proteases released by cells into the cell culture supernatant during the protein production process[89,106,107]. According to several reports, the culture media components have different effects on product fragmentation. Trace elements, including manganese, zinc, and cobalt, decrease LMW formation[108], while copper increases the LMW formation[19,90]. Also, other media components, such as EDTA[19,90,108] and cysteine[17] decrease product fragmentation.

#### Aggregation during cell culture

The control of the produced aggregates level during the cell culture process is possible. This control is accomplished by carefully choosing the proper cell line and improving cell culture conditions, such as media components that affect media osmolality and conductivity, feeding strategy, temperature, and pH[109]. A lower quantity of aggregates in the secreted protein was observed when media pH and osmolarity were increased in cells cultured in a hollow fibre bioreactor[110]. Cromwell *et al*.[18] studied the effect of cell culture temperature on aggregate formation during the culture. They indicated that the higher levels of aggregates were observed when the protein was held in the culture medium at a high temperature for a longer time. Different effects of media components on product aggregation have been reported. Different reducing and oxidizing substances containing glutathione, cysteine, and copper decrease the protein aggregate formation in CHO cell culture harvests[17]. Sodium chloride also decreases the aggregate amount[111].

#### Analytical methods for the detection of fragmentation and aggregation of mAbs

The analytical methods used to detect fragmentation and aggregation can be divided into two groups based on their separation mode: (1) The methods in which the separation is based on the size of the molecule, such as size-exclusion chromatography, sodium dodecyl sulfate-polyacrylamide gel electrophoresis, and CE with SDS. (2) The methods in which the separation is based on the chemistry of amino acid side chains such as cation exchange chromatography. While the mentioned methods are usually used to monitor and quantify protein fragmentation and aggregation, the identification of the exact cleavage site is performed using mass spectrometry[112,113].

Here, we explained the main quality attributes of recombinant mAbs, which can be altered during cell culture media optimization. In cell culture media optimization, the challenge is to increase the yield with the desired quality of the product by the addition of appropriate components at the correct concentration. Published data show that quality engineering could be performed by media design which is a rational strategy to considerably control the main quality attributes and function of mAbs. Therefore, to reach a recombinant mAb with the desired quality, the analysis of main quality attributes by appropriate analytical methods during the process development is necessary and inevitable.

## References

[1] Chugh PK, Roy V (2014). Biosimilars: current scientific and regulatory considerations. Current clinical pharmacology.

[2] Kenett RS, Kenett DA (2008). Quality by design applications in biosimilar pharmaceutical products. Accreditation and quality assurance.

[3] Rathore AS, Winkle H (2009). Quality by design for biopharmaceuticals. Nature biotechnology.

[4] Lawrence XYU (2008). Pharmaceutical quality by design: product and process development, understanding, and control. Pharmaceutical research.

[5] Mahboudi F, Abolhassan MR, Azarpanah A, Aghajani-Lazarjani H, Sadeghi-Haskoo MA, Maleknia S, Vaziri B (2013). The role of different supplements in expression level of monoclonal antibody against human CD20. Avicenna journal of medical biotechnology.

[6] Zarei N, Vaziri B, Shokrgozar MA, Mahdian R, Fazel R, Khalaj V (2014). High efficient expression of a functional humanized single-chain variable fragment (scFv) antibody against CD22 in Pichia pastoris. Applied microbiology and biotechnology.

[7] Ivarsson M, Villiger TK, Morbidelli M, Soos M (2014). Evaluating the impact of cell culture process parameters on monoclonal antibody N-glycosylation. Journal of biotechnology.

[8] Du Z, Treiber D, McCarter JD, Fomina-Yadlin D, Saleem RA, McCoy RE, Zhang Y, Tharmalingam T, Leith M, Follstad BD, Dell B, Grisim B, Zupke C, Heath C, Morris AE, Reddy P (2015). Use of a small molecule cell cycle inhibitor to control cell growth and improve specific productivity and product quality of recombinant proteins in CHO cell cultures. Biotechnology and bioengineering.

[9] Schmelzer AE, Miller WM (2002). Hyperosmotic stress and elevated pCO2 alter monoclonal antibody charge distribution and monosaccharide content. Biotechnology progress.

[10] Meuwly F, Weber U, Ziegler T, Gervais A, Mastrangeli R, Crisci C, Rossi M, Bernard A, von Stockar U, Kadouri A (2006). Conversion of a CHO cell culture process from perfusion to fed-batch technology without altering product quality. Journal of biotechnology.

[11] Kremkow B, Lee KH (2013). Next-generation sequencing technologies and their potential impact on CHO cell-based biomanufacturing. Pharmaceutical bioprocessing.

[12] Butler M, Meneses-Acosta A (2012). Recent advances in technology supporting biopharmaceutical production from mammalian cells. Applied microbiology and biotechnology.

[13] Zhu J (2012). Mammalian cell protein expression for biopharmaceutical production. Biotechnology advances.

[14] Gramer MJ (2014). Product quality considerations for mammalian cell culture process development and manufacturing. Advances in biochemical engineering/biotechnology.

[15] Gramer MJ, Eckblad JJ, Donahue R, Brown J, Shultz C, Vickerman K, Priem P, van den Bremer ET, Gerritsen J, van Berkel PH (2011). Modulation of antibody galactosylation through feeding of uridine, manganese chloride, and galactose. Biotechnology and bioengineering.

[16] Luo J, Zhang J, Ren D, Tsai WL, Li F, Amanullah A, Hudson T (2012). Probing of C-terminal lysine variation in a recombinant monoclonal antibody production using Chinese hamster ovary cells with chemically defined media. Biotechnology and bioengineering.

[17] Jing Y, Borys M, Nayak S, Egan S, Qian Y, Pan SH, Li ZJ (2012). Identification of cell culture conditions to control protein aggregation of IgG fusion proteins expressed in Chinese hamster ovary cells. Process Biochemistry.

[18] Cromwell ME, Hilario E, Jacobson F (2006). Protein aggregation and bioprocessing. The AAPS journal.

[19] Trexler-Schmidt M, Sargis S, Chiu J, Sze-Khoo S, Mun M, Kao YH, Laird MW (2010). Identification and prevention of antibody disulfide bond reduction during cell culture manufacturing. Biotechnology and bioengineering.

[20] Khawli LA, Goswami S, Hutchinson R, Kwong ZW, Yang J, Wang X, Yao Z, Sreedhara A, Cano T, Tesar D, Nijem I, Allison DE, Wong PY, Kao YH, Quan C, Joshi A, Harris RJ, Motchnik P (2010). Charge variants in IgG1: Isolation, characterization *in vitro* binding properties and pharmacokinetics in rats. MAbs.

[21] Boswell CA, Tesar DB, Mukhyala K, Theil FP, Fielder PJ, Khawli LA (2010). Effects of charge on antibody tissue distribution and pharmacokinetics. Bioconjugate chemistry.

[22] Bumbaca D, Boswell CA, Fielder PJ, Khawli LA (2012). Physiochemical and biochemical factors influencing the pharmacokinetics of antibody therapeutics. The AAPS journal.

[23] Wang W, Wang EQ, Balthasar JP (2008). Monoclonal antibody pharmacokinetics and pharmacodynamics. Clinical pharmacology and therapeutics.

[24] Brooks SA (2006). Protein glycosylation in diverse cell systems: implications for modification and analysis of recombinant proteins. Expert review of proteomics.

[25] Apweiler R, Hermjakob H, Sharon N (1999). On the frequency of protein glycosylation, as deduced from analysis of the SWISS-PROT database. Biochimica et Biophysica Acta (BBA)-General Subjects.

[26] Liu H, Gaza-Bulseco G, Faldu D, Chumsae C, Sun J (2008). Heterogeneity of monoclonal antibodies. Journal of pharmaceutical sciences.

[27] Pacis E, Yu M, Autsen J, Bayer R, Li F (2011). Effects of cell culture conditions on antibody N-linked glycosylation—what affects high mannose 5 glycoform. Biotechnology and bioengineering.

[28] Rouiller Y, Périlleux A, Marsaut M, Stettler M, Vesin MN, Broly H (2012). Effect of hydrocortisone on the production and glycosylation of an Fc-fusion protein in CHO cell cultures. Biotechnology progress.

[29] Chee Furng Wong D, Tin Kam Wong K, Tang Goh L, Kiat Heng C, Gek Sim Yap M (2005). Impact of dynamic online fed-batch strategies on metabolism, productivity and N-glycosylation quality in CHO cell cultures. Biotechnology and bioengineering.

[30] Konno Y, Kobayashi Y, Takahashi K, Takahashi E, Sakae S, Wakitani M, Yamano K, Suzawa T, Yano K, Ohta T, Koike M, Wakamatsu K, Hosoi S (2012). Fucose content of monoclonal antibodies can be controlled by culture medium osmolality for high antibody-dependent cellular cytotoxicity. Cytotechnology.

[31] Yang J-M, Ai J, Bao Y, Yuan Z, Qin Y, Xie YW, Tao D, Fu D, Peng Y (2014). Investigation of the correlation between charge and glycosylation of IgG1 variants by liquid chromatography–mass spectrometry. Analytical biochemistry.

[32] Jefferis R (2009). Glycosylation as a strategy to improve antibody-based therapeutics. Nature reviews drug discovery.

[33] Goetze AM, Liu YD, Zhang Z, Shah B, Lee E, Bondarenko PV, Flynn GC (2011). High-mannose glycans on the Fc region of therapeutic IgG antibodies increase serum clearance in humans. Glycobiology.

[34] Yu M, Brown D, Reed C, Chung S, Lutman J, Stefanich E, Wong A, Stephan JP, Bayer R (2012). Production, characterization and pharmacokinetic properties of antibodies with N-linked Mannose-5 glycans. MAbs.

[35] Zhong X, Cooley C, Seth N, Juo SZ, Presman E, Resendes N, Kumar R, Allen M, Mosyak L, Stahi M, Somers W, Kriz R (2012). Engineering novel Lec1 glycosylation mutants in CHO–DUKX cells: Molecular insights and effector modulation of N-acetylglucosaminyltransferase I. Biotechnology and bioengineering.

[36] Torkashvand F, Vaziri B, Maleknia S, Heydari A, Vossoughi M, Davami F, Mahboudi F (2015). Designed amino acid feed in improvement of production and quality targets of a therapeutic monoclonal antibody. PLoS One.

[37] Crowell CK, Grampp GE, Rogers GN, Miller J, Scheinman RI (2007). Amino acid and manganese supplementation modulates the glycosylation state of erythropoietin in a CHO culture system. Biotechnology and bioengineering.

[38] Kaufman RJ, Swaroop M, Murtha-Riel P (1994). Depletion of manganese within the secretory pathway inhibits O-linked glycosylation in mammalian cells. Biochemistry.

[39] Wong NS, Wati L, Nissom PM, Feng HT, Lee MM, Yap MG (2010). An investigation of intracellular glycosylation activities in CHO cells: effects of nucleotide sugar precursor feeding. Biotechnology and bioengineering.

[40] Nyberg GB, Balcarcel RR, Follstad BD, Stephanopoulos G, Wang DI (1999). Metabolic effects on recombinant interferon-? glycosylation in continuous culture of Chinese hamster ovary cells. Biotechnology and bioengineering.

[41] Jayme D, Watanabe T, Shimada T (1997). Basal medium development for serum-free culture: a historical perspective. Cytotechnology.

[42] Liu B, Spearman M, Doering J, Lattová E, Perreault H, Butler M (2014). The availability of glucose to CHO cells affects the intracellular lipid-linked oligosaccharide distribution, site occupancy and the N-glycosylation profile of a monoclonal antibody. Journal of biotechnology.

[43] Seo JS, Min BS, Kim YJ, Cho JM, Baek E, Cho MS, Lee GM (2014). Effect of glucose feeding on the glycosylation quality of antibody produced by a human cell line, F2N78, in fed-batch culture. Applied microbiology and biotechnology.

[44] Hayter PM, Curling E, Baines AJ, Jenkins N, Salmon I, Strange PG, Tong JM, Bull AT (1992). Glucose-limited chemostat culture of chinese hamster ovary cells producing recombinant human interferon-?. Biotechnology and bioengineering.

[45] Cruz HJ, Peixoto CM, Nimtz M, Alves PM, Dias EM, Moreira JL, Carrondo MJ (2000). Metabolic shifts do not influence the glycosylation patterns of a recombinant fusion protein expressed in BHK cells. Biotechnology and bioengineering.

[46] Raju TS, Jordan RE (2012). Galactosylation variations in marketed therapeutic antibodies. MAbs.

[47] Anumula KR, Dhume ST (1998). High resolution and high sensitivity methods for oligosaccharide mapping and characterization by normal phase high performance liquid chromatography following derivatization with highly fluorescent anthranilic acid. Glycobiology.

[48] Kamoda S, Nomura C, Kinoshita M, Nishiura S, Ishikawa R, Kakehi K, Kawasaki N, Hayakawa T (2004). Profiling analysis of oligosaccharides in antibody pharmaceuticals by capillary electrophoresis. Journal of chromatography.

[49] Guile GR, Rudd PM, Wing DR, Prime SB, Dwek RA (1996). A rapid high-resolution high-performance liquid chromatographic method for separating glycan mixtures and analyzing oligosaccharide profiles. Analytical biochemistry.

[50] Spellman MW (1990). Carbohydrate characterization of recombinant glycoproteins of pharmaceutical interest. Analytical chemistry.

[51] Townsend RR, Hardy M, Lee YC (1988). Separation of oligosaccharides using high-performance anion-exchange chromatography with pulsed amperometric detection. Methods in enzymology.

[52] Kamoda S, Ishikawa R, Kakehi K (2006). Capillary electrophoresis with laser-induced fluorescence detection for detailed studies on N-linked oligosaccharide profile of therapeutic recombinant monoclonal antibodies. Journal of chromatography a.

[53] Reusch D, Haberger M, Maier B, Maier M, Kloseck R, Zimmermann B, Hook M, Szabo Z, Tep S, Wegstein J, Alt N, Bulau P, Wuhrer M (2015). Comparison of methods for the analysis of therapeutic immunoglobulin G Fc-glycosylation profiles—part 1: Separation-based methods.

[54] Kaschak T, Boyd D, Lu F, Derfus G, Kluck B, Nogal B, Emery C, Summers C, Zheng K, Bayer R, Amanullah A, Yan B (2011). Characterization of the basic charge variants of a human IgG1: effect of copper concentration in cell culture media. MAbs.

[55] Khawli LA, Glasky MS, Alauddin MM, Epstein AL (1996). Improved tumor localization and radioimaging with chemically modified monoclonal antibodies. Cancer biotherapy and radiopharmaceuticals.

[56] Perera RM, Zoncu R, Johns TG, Pypaert M, Lee FT, Mellman I, Old LJ, Toomre DK, Scott AM (2007). Internalization, intracellular trafficking, biodistribution of monoclonal antibody 806: a novel anti-epidermal growth factor receptor antibody. Neoplasia.

[57] Lee HJ, Pardridge WM (2003). Monoclonal antibody radiopharmaceuticals: cationization, pegylation, radiometal chelation, pharmacokinetics, and tumor imaging. Bioconjugate chemistry.

[58] Khawli LA, Mizokami MM, Sharifi J, Hu P, Epstein AL (2002). Pharmacokinetic characteristics and biodistribution of radioiodinated chimeric TNT-1,-2, and-3 monoclonal antibodies after chemical modification with biotin. Cancer biotherapy and radiopharmaceuticals.

[59] Xie P, Niu H, Chen X, Zhang X, Miao S, Deng X, Liu X, Tan WS, Zhou Y, Fan L (2016). Elucidating the effects of pH shift on IgG1 monoclonal antibody acidic charge variant levels in Chinese hamster ovary cell cultures. Applied microbiology and biotechnology.

[60] Hossler P, Wang M, McDermott S, Racicot C, Chemfe K, Zhang Y, Chumsae C, Manuilov A (2015). Cell culture media supplementation of bioflavonoids for the targeted reduction of acidic species charge variants on recombinant therapeutic proteins. Biotechnology progress.

[61] Kishishita S, Nishikawa T, Shinoda Y, Nagashima H, Okamoto H, Takuma S, Aoyagi H (2015). Effect of temperature shift on levels of acidic charge variants in IgG monoclonal antibodies in Chinese hamster ovary cell culture. Journal of bioscience and bioengineering.

[62] Neill A, Nowak C, Patel R, Ponniah G, Gonzalez N, Miano D, Liu H (2015). Characterization of recombinant monoclonal antibody charge variants using offgel fractionation, weak anion exchange chromatography, and mass spectrometry. Analytical chemistry.

[63] Lyubarskaya Y, Houde D, Woodard J, Murphy D, Mhatre R (2006). Analysis of recombinant monoclonal antibody isoforms by electrospray ionization mass spectrometry as a strategy for streamlining characterization of recombinant monoclonal antibody charge heterogeneity. Analytical biochemistry.

[64] Beck A, Bussat MC, Zorn N, Robillard V, Klinguer-Hamour C, Chenu S, Goetsch L, Corvaïa N, Van Dorsselaer A, Haeuw JF (2005). Characterization by liquid chromatography combined with mass spectrometry of monoclonal anti-IGF-1 receptor antibodies produced in CHO and NS0 cells. Journal of chromatography B.

[65] Antes B, Amon S, Rizzi A, Wiederkum S, Kainer M, Szolar O, Fido M, Kircheis R, Nechansky A (2007). Analysis of lysine clipping of a humanized Lewis-Y specific IgG antibody and its relation to Fc-mediated effector function. Journal of chromatography b.

[66] Santora LC, Stanley K, Krull IS, Grant K (2006). Characterization of maleuric acid derivatives on transgenic human monoclonal antibody due to post-secretional modifications in goat milk. Biomedical chromatography.

[67] Yan B, Steen S, Hambly D, Valliere-Douglass J, Vanden Bos T, Smallwood S, Yates Z, Arroll T, Han Y, Gadgil H, Latypov RF, Wallace A, Lim A, Kleemann GR, Wang W, Balland A (2009). Succinimide formation at Asn 55 in the complementarity determining region of a recombinant monoclonal antibody IgG1 heavy chain. Journal of pharmaceutical sciences.

[68] Du Y, Walsh A, Ehrick R, Xu W, May K, Liu H (2012). Chromatographic analysis of the acidic and basic species of recombinant monoclonal antibodies. Mabs.

[69] Kim J, Jones L, Taylor L, Kannan G, Jackson F, Lau H, Latypov RF, Bailey B (2010). Characterization of a unique IgG1 mAb CEX profile by limited Lys-C proteolysis/CEX separation coupled with mass spectrometry and structural analysis. Journal of chromatography b.

[70] Vlasak J, Bussat MC, Wang S, Wagner-Rousset E, Schaefer M, Klinguer-Hamour C, Kirchmeier M, Corvaïa N, Ionescu R, Beck A (2009). Identification and characterization of asparagine deamidation in the light chain CDR1 of a humanized IgG1 antibody. Analytical biochemistry.

[71] Harris RJ, Kabakoff B, Macchi FD, Shen FJ, Kwong M, Andya JD, Shire SJ, Bjork N, Totpal K, Chen AB (2001). Identification of multiple sources of charge heterogeneity in a recombinant antibody. Journal of chromatography b: biomedical sciences and applications.

[72] Huang L, Lu J, Wroblewski VJ, Beals JM, Riggin RM (2005). *In vivo* deamidation characterization of monoclonal antibody by LC/MS/MS. Analytical chemistry.

[73] Zheng JY, Janis LJ (2006). Influence of pH, buffer species, and storage temperature on physicochemical stability of a humanized monoclonal antibody LA298. International journal of pharmaceutics.

[74] Perkins M, Theiler R, Lunte S, Jeschke M (2000). Determination of the origin of charge heterogeneity in a murine monoclonal antibody. Pharmaceutical research.

[75] Moorhouse K, Nashabeh W, Deveney J, Bjork NS, Mulkerrin MG, Ryskamp T (1997). Validation of an HPLC method for the analysis of the charge heterogeneity of the recombinant monoclonal antibody IDEC-C2B8 after papain digestion. Journal of pharmaceutical and biomedical analysis.

[76] Santora LC, Krull IS, Grant K (1999). Characterization of recombinant human monoclonal tissue necrosis factor-a antibody using cation-exchange HPLC and capillary isoelectric focusing. Analytical biochemistry.

[77] Dick LW, Qiu D, Mahon D, Adamo M, Cheng KC (2008). C-terminal lysine variants in fully human monoclonal antibodies: Investigation of test methods and possible causes. Biotechnology and bioengineering.

[78] Lau H, Pace D, Yan B, McGrath T, Smallwood S, Patel K, Park J, Park SS, Latypov RF (2010). Investigation of degradation processes in IgG1 monoclonal antibodies by limited proteolysis coupled with weak cation-exchange HPLC. Journal of chromatography b.

[79] Zhang X, Sun Y-T, Tang H, Fan L, Hu D, Liu J, Liu X, Tan WS (2015). Culture temperature modulates monoclonal antibody charge variation distribution in Chinese hamster ovary cell cultures. Biotechnology letters.

[80] Wakankar AA, Borchardt RT (2006). Formulation considerations for proteins susceptible to asparagine deamidation and aspartate isomerization. Journal of pharmaceutical science.

[81] Zhou S, Zhang B, Sturm E, Teagarden DL, Schöneich C, Kolhe P, Lewis LM, Muralidhara BK, Singh SK (2010). Comparative evaluation of disodium edetate and diethylenetriaminepentaacetic acid as iron chelators to prevent metal-catalyzed destabilization of a therapeutic monoclonal antibody. Journal of pharmaceutical sciences.

[82] Hong JK, Lee SM, Kim KY, Lee GM (2014). Effect of sodium butyrate on the assembly, charge variants, and galactosylation of antibody produced in recombinant Chinese hamster ovary cells. Applied microbiology and biotechnology.

[83] Hossler P, Wang M, McDermott S, Racicot C, Chemfe K, Zhang Y, Chumsae C, Manuilov A (2015). Cell culture media supplementation of bioflavonoids for the targeted reduction of acidic species charge variants on recombinant therapeutic proteins. Biotechnology progress.

[84] Tsai PK, Bruner MW, Irwin JI, Ip CC, Oliver CN, Nelson RW, Volkin DB, Middaugh CR (1993). Origin of the isoelectric heterogeneity of monoclonal immunoglobulin h1B4. Pharmaceutical research.

[85] Quan C, Alcala E, Petkovska I, Matthews D, Canova-Davis E, Taticek R, Ma S (2008). A study in glycation of a therapeutic recombinant humanized monoclonal antibody: where it is, how it got there, and how it affects charge-based behavior. Analytical biochemistry.

[86] Johnson KA, Paisley-Flango K, Tangarone BS, Porter TJ, Rouse JC (2007). Cation exchange–HPLC and mass spectrometry reveal C-terminal amidation of an IgG1 heavy chain. Analytical biochemistry.

[87] Pristatsky P, Cohen SL, Krantz D, Acevedo J, Ionescu R, Vlasak J (2009). Evidence for trisulfide bonds in a recombinant variant of a human IgG2 monoclonal antibody. Analytical chemistry.

[88] Rosenberg AS (2006). Effects of protein aggregates: an immunologic perspective. The AAPS journal.

[89] Gao SX, Zhang Y, Stansberry-Perkins K, Buko A, Bai S, Nguyen V, Brader M (2011). Fragmentation of a highly purified monoclonal antibody attributed to residual CHO cell protease activity. Biotechnology and bioengineering.

[90] Vlasak J, Ionescu R (2011). Fragmentation of monoclonal antibodies. MAbs.

[91] Jenkins N, Murphy L, Tyther R (2008). Post-translational modifications of recombinant proteins: significance for biopharmaceuticals. Molecular biotechnology.

[92] Eon-Duval A, Broly H, Gleixner R (2012). Quality attributes of recombinant therapeutic proteins: an assessment of impact on safety and efficacy as part of a quality by design development approach. Biotechnology progress.

[93] del Val IJ, Kontoravdi C, Nagy JM (2010). Towards the implementation of quality by design to the production of therapeutic monoclonal antibodies with desired glycosylation patterns. Biotechnology progress.

[94] Vázquez-Rey M, Lang DA (2011). Aggregates in monoclonal antibody manufacturing processes. Biotechnology and bioengineering.

[95] Philo JS, Arakawa T (2009). Mechanisms of protein aggregation. Current pharmaceutical biotechnology.

[96] Maas C, Hermeling S, Bouma B, Jiskoot W, Gebbink MF (2007). A role for protein misfolding in immunogenicity of biopharmaceuticals. Journal of biological chemistry.

[97] Perchiacca JM, Tessier PM (2012). Engineering aggregation-resistant antibodies. Annual review of chemical and biomolecular engineering.

[98] Paul R, Graff-Meyer A, Stahlberg H, Lauer ME, Rufer AC, Beck H, Briguet A, Schnaible V, Buckel T, Boeckle S (2012). Structure and function of purified monoclonal antibody dimers induced by different stress conditions. Pharmaceutical research.

[99] Dengl S, Wehmer M, Hesse F (2013). Aggregation and chemical modification of monoclonal antibodies under upstream processing conditions. Pharmaceutical research.

[100] Wang W (2005). Protein aggregation and its inhibition in biopharmaceutics. International journal of pharmaceutics.

[101] Wang W, Nema S, Teagarden D (2010). Protein aggregation—Pathways and influencing factors. International journal of pharmaceutics.

[102] Patel J, Kothari R, Tunga R, Ritter NM, Binita ST (2011). Stability considerations for biopharmaceuticals, Part 1: Overview of protein and peptide degradation pathways. Bioprocess International.

[103] Matsuoka T, Tomita S, Hamada H, Shiraki K (2007). Amidated amino acids are prominent additives for preventing heat-induced aggregation of lysozyme. Journal of bioscience and bioengineering.

[104] Okanojo M, Shiraki K, Kudou M, Nishikori S, Takagi M (2005). Diamines prevent thermal aggregation and inactivation of lysozyme. Journal of bioscience and bioengineering.

[105] Arakawa T, Timasheff S (1985). The stabilization of proteins by osmolytes. Biophysical Journal.

[106] Robert F, Bierau H, Rossi M, Agugiaro D, Soranzo T, Broly H, Mitchell-Logean C (2009). Degradation of an Fc-fusion recombinant protein by host cell proteases: Identification of a CHO cathepsin D protease. Biotechnology and bioengineering.

[107] Ouellette D, Alessandri L, Piparia R, Aikhoje A, Chin A, Radziejewski C, Correia I (2009). Elevated cleavage of human immunoglobulin gamma molecules containing a lambda light chain mediated by iron and histidine. Analytical biochemistry.

[108] Kao YH, Hewitt DP, Trexler-Schmidt M, Laird MW (2010). Mechanism of antibody reduction in cell culture production processes. Biotechnology and bioengineering.

[109] Gabrielson JP, Brader ML, Pekar AH, Mathis KB, Winter G, Carpenter JF, Randolph TW (2007). Quantitation of aggregate levels in a recombinant humanized monoclonal antibody formulation by size-exclusion chromatography, asymmetrical flow field flow fractionation, and sedimentation velocity. Journal of pharmaceutical sciences.

[110] Franco R, Daniela G, Fabrizio M, Ilaria G, Detlev H (1999). Influence of osmolarity and pH increase to achieve a reduction of monoclonal antibodies aggregates in a production process. Cytotechnology.

[111] Ju HK, Hwang SJ, Jeon CJ, Lee GM, Yoon SK (2009). Use of NaCl prevents aggregation of recombinant COMP–Angiopoietin-1 in Chinese hamster ovary cells. Journal of biotechnology.

[112] Davagnino J, Wong C, Shelton L, Mankarious S (1995). Acid hydrolysis of monoclonal antibodies. Journal of immunological methods.

[113] Smith MA, Easton M, Everett P, Lewis G, Payne M, Riveros-Moreno V, Allen G (1996). Specific cleavage of immunoglobulin G by copper ions. International journal of peptide and protein research.

